# Boğaziçi University distributed denial of service dataset

**DOI:** 10.1016/j.dib.2020.106187

**Published:** 2020-08-17

**Authors:** Derya Erhan, Emin Anarım

**Affiliations:** Boğaziçi University Electrical and Electronics Engineering, İstanbul, Turkey

**Keywords:** DDoS, UDP flood, TCP flood, Intrusion Detection, Network Security

## Abstract

Distributed Denial of Service (DDoS) attacks is one of the most troublesome intrusions for online services on the internet. In general DDoS attacks are divided into two categories as bandwidth depletion and resource depletion attacks. We generate resource depletion-type DDoS attacks on the campus network of Boğaziçi University and recorded the ongoing traffic from the backbone router's mirrored port. We generate TCP SYN, and UDP flooding packets using Hping3 traffic generator software by flooding. This dataset includes attack-free user traffic and attack traffic, which is suitable for evaluating network-based DDoS detection methods. Attacks are towards one victim server connected to the backbone router of the campus. Attack packets have randomly generated spoofed source IP addresses. We removed payloads of packets and anonymized the source IP addresses of legitimate users for the confidentiality of legitimate users.

**Specifications Table**

**Table:** 

**Subject**	Computer Science:: Computer Networks and Communications, Computer Vision and Pattern Recognition, Information Systems.
**Specific subject area**	Resource depletion type DDoS attacks, TCP SYN flood, and UDP flood.
**Type of data**	Table
**How data were acquired**	From network switch using Wireshark [1] software.
**Data format**	Raw
	Anonymised
**Parameters for data collection**	Data Collection occurred in Boğaziçi University Campus network. Flood packets are generated using three computers via hping3 software. Attack-free data in the dataset is the traffic of more than 4000 real internet users. The payloads of packets are removed, and the IP addresses of the users are changed to protect the confidentiality of users and the university.
**Description of data collection**	Data is collected from mirrored backbone switch port using Wireshark software in .pcap file format. These files then converted to .cvs format, and packet payloads removed for anonymization.
**Data source location**	Boğaziçi University, İstanbul, Turkey.
**Data accessibility**	DOI: http://dx.doi.org/10.17632/mfnn9bh42m.1#file-ba7d3a46-1dc3-452e-aeac-26d909389b29

**Value of the Data**

• Dataset provides aspects of DDoS attacks, including network-based probing of two-way legitimate user traffic mixed up with DDoS packets. Besides, it includes attacks of different intensities, to help researchers train and evaluate their intrusion detection approaches for different attack densities.
• These datasets provide a general understanding of resource depletion type DDoS attacks collected from the backbone router of campus networks. These datasets are suitable for developing and evaluating network-based attack detection methodologies. Boğaziçi University DDoS (BOUN DDoS) Dataset has been already used in some academic papers [1], [2], [3], [4], [5], [6], [7], [8], [9]
• Unlike other publicly available DDoS datasets [10,11], the BOUN dataset includes legitimate background internet traffic mixed with DDoS attack traffic. In addition, the BOUN datasets provide easier simulation and analysis because of small file sizes and fewer packets compared to other datasets [10,11,12].
• Attack and legitimate traffic packets can easily be separated from each other using destination IP addresses of packets. Attack-free packets in the datasets can be used for traffic analysis, or combined methods with another attack dataset can be evaluated [13].
• Datasets are given in comma-separated file format, including header information of packets to help researchers easily import datasets in different research software platforms.

## 1 Data description

**Fig. 1:**
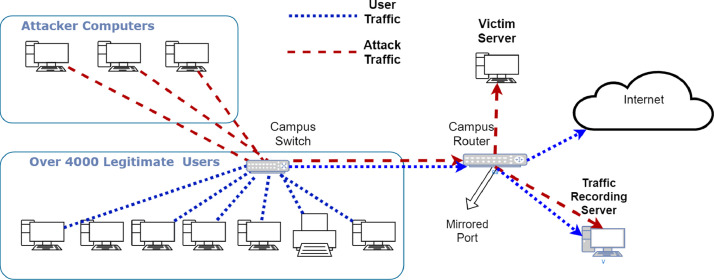
Network topology of BOUN DDoS dataset traffic generation and recording.

The design concept of Network-based intrusion detection systems is detecting attacks from networks end, on the router, or on the backbone switch. This dataset is produced for the evaluation of network-based intrusion detection methods. In the network topology shown in Fig. 1, the traffic is taken from campus routers port by mirroring method. The mirroring operation on routers interfaces provides our traffic recording server the exact copies of incoming and outgoing packets flowing through the mirrored interface. Traffic is recorded and converted to .csv file format using Wireshark software.

The dataset includes two different attack scenarios. In both situations, randomly generated spoofed destination IP addresses are used in a flooding manner. For TCP flood attacks, TCP port 80 is used as the destination port. All of the datasets lasted 8 min. In each of them, 80 s waiting period, then 20 s attack period is practiced. Different packet rates are used to let researchers evaluate their detection methods concerning different packet rates.

The TCP SYN Flood and UDP flood datasets include attack rates of 1000, 1500, 2000, and 2500 packets/second, respectively. The topology of the network for obtaining an attack dataset is given in Fig. 1. Both legitimate and DDoS attack traffics mirrored to the recording server.

Attack packets can be distinguished from attack-free packets using the destination IP address of packets. The victim IP address is 10.50.199.86.

Fig. 1 shows the network topology used in the generation of the dataset. We carried out the TCP SYN flood and UDP flood attacks towards a server connected to the campus backbone. Over 4000 active internet user traffic was flowing over the campus router simultaneously to the attack traffic.

We used the hping3 software installed on 3 computers for attacks. Attack packets contain spoofed source IP addresses. Since the source IP addresses of the attack packets are generated randomly and uniquely, it appears as attacks come from many different sources when viewed from the routers port. In other words, the attack packets in the dataset come from multiple sources.

Datasets are given as two tables in the comma-separated value (csv) file format. The names of the files are BOUN_TCP_Anon.csv corresponding to TCP SYN flood attacks, and BOUN_UDP_Anon.csv corresponding to the UDP flood attack dataset. The tables in the files of the dataset have the following columns:

1 Time: Time values start from zero and have a resolution of 0.000001 s. Time values are expressed in seconds.
2 Frame Number: Frame number is simply the incremental count of packets in the dataset.
3 Frame_length: Frame length is the length of that packet in bytes.
4 Source_ip: Source IP address of the packet.
5 Destination_IP: Destination Ip address of the packet.
6 Source_Port: Source TCP port of the packet. If it is not a TCP packet, this field is empty.
7 Destination_Port: Destination TCP port of the packet. If it is not a TCP packet, this field is empty
8 SYN: This value is "Set" if the packet is a TCP packet and its SYN flag is equal to one, it is equal to "Not Set" if the packet is a TCP packet and its SYN flag is equal to zero. If the packet is not a TCP packet, this field is empty.
9 ACK: This value is "Set" if the packet is a TCP packet and its ACK flag is equal to one, it is equal to "Not Set" if the packet is a TCP packet and its ACK flag is equal to zero. If the packet is not a TCP packet, this field is empty.
10 RST: This value is "Set" if the packet is a TCP packet and its RST flag is equal to one, it is equal to "Not Set" if the packet is a TCP packet and its RST flag is equal to zero. If the packet is not a TCP packet, this field is empty.
11 TTL: Time to live value of the packets.
12 TCP_Protocol: This value can be TCP or UDP if the packet belongs to a transport layer IP protocol. Else this value can have different values.

Table 1 and Table 2 gives some statistics and information about attacks in datasets. Each attack dataset contains 4 attack instances. The columns of tables are explained as follows:

• Attack Period: There are 4 attack periods for TCP SYN and UDP flood datasets.
• Start Time: Each dataset timer starts from zero. The start time column corresponds to the start time of the attack in seconds.
• End Time: The end time of the attacks in seconds.
• Start Frame: The frame number of the first packet of the attack.
• End Frame: The frame number of the last packet of the attack.
• Attack Packets: The number of attack packets in the attack instance.
• Legitimate packets: The number of attack-free packets in the attack instance.
• Density: The ratio of the number of attack packets to the number of attack-free packets. This ratio is calculated in the time window where the attack packet exists.

**Table 1:** Information about attack instances in BOUN TCP SYN Flood attack dataset.

Attack period	Start Time (*sec*)	End time (*sec*)	Start Frame	End Frame	Attack Packets	Legitimate Packets	Density
1	80.22269	102.20233	2,335,362	2,335,362	19,035	370,746	0.05134
2	180.17426	203.08441	4,240,070	4,240,070	27,121	428,168	0.06334
3	279.97402	301.79111	5,959,329	5,959,329	35,936	352,296	0.10201
4	380.10981	402.35755	7,885,602	7,885,602	43,465	401,553	0.10824

**Table 2:** Information about attack instances in BOUN UDP Flood attack dataset.

Attack Period	Start Time (*sec*)	End time (*sec*)	Start Frame	End Frame	Attack Packets	Legitimate Packets	Density
1	80.87054	102.68198	1,354,950	1,354,950	37,216	268,882	0.13841
2	180.94241	203.55186	2,931,244	2,931,244	55,029	337,036	0.16327
3	280.59444	303.16265	4,702,829	4,702,829	75,023	393,450	0.19068
4	381.01394	403.65057	6,513,625	6,513,625	93,378	404,330	0.23095

## 2 Experimental design, materials and methods

We used the same network topology shown in Fig. 1 to create the UDP and TCP SYN flood datasets. The setup differs only in the generated attack packets for UDP and TCP SYN flood attack datasets. We used hping3 software to generate attack packets with randomly generated spoofed source IP addresses.

Network-based intrusion detection systems aim to detect intrusions by monitoring traffic to and from all devices. They perform detection by analyzing all traffic passing through the gateway of the user networks. They are generally connected to the gateway of the network or the backbone router.

We produced the BOUN DDoS dataset to evaluate network-based intrusion detection approaches. We recorded the network traffic from the mirrored router port. Port mirroring on the backbone router sends a copy of all network packets seen on the mirrored router port to another interface for monitoring purposes.

Wireshark software running on a server running with windows processing system was used to record the traffic. Traffic is initially saved in .pcap file format and then converted into the .csv file format to make it available to use in research software applications. Payloads of packets are deleted, and A-class virtual IP addresses replace source IP addresses using text editing software to preserve the confidentiality of end-users.

## Ethics statement

This work doesn't include any human subject and animal experiments. In addition, data is anonymized, and the payload of the packets is removed in order to prevent the confidentiality of users.

## Declaration of Competing Interest

The authors declare that they have no known competing for financial interests or personal relationships that could have appeared to influence the work reported in this paper.

## Appendix Supplementary materials

Image, application 1
